# Diseases of the Intestines and Peritoneum

**Published:** 1879-07

**Authors:** 


Diseases of the Intestines and Peritoneum. By Jno. Syer Bristowe, M D , J. R. Wardell, M.D., J. W. Bigbie, M.D., S. O. Habeshon, M. D., T. B. Curling, M.D., Wm. Wood & Co., N. Y., 1879.

This volume has kindly been sent to us for review by the enterprising publishers, Messrs. Wood & Co., and is a worthy companion to those already published of the series of "Library of Standard Authors." The subjects treated of are of wide spread interest and importance, and coming from authors of recognized ability as they do, cannot fail to meet a good reception. Diseases of the intestines, anus and peritoneum are elaborately treated of, and their various pathological relations, with their therapeutical indications presented.

